# Positive diagnostic positional tests in BPPV are higher in the morning than in the afternoon: a retrospective study

**DOI:** 10.3389/fneur.2025.1689672

**Published:** 2025-10-07

**Authors:** Qiuping Lu, Ting Zhan, Jiabao Xu, Huihong Chen, Jinzhang Zhou, Huaifeng Liu, Tinglong Yang, Michael Strupp, Biru Zhang

**Affiliations:** ^1^Department of Otorhinolaryngology, Head and Neck Surgery, The Eighth Affiliated Hospital, Southern Medical University (The First People's Hospital of Shunde, Foshan), Foshan, China; ^2^Department of Otolaryngology-Head and Neck Surgery, Xiangya Hospital, Central South University, Changsha, Hunan, China; ^3^Ning Xia Center for Disease Control and Prevention, Ningxia, China; ^4^Department of Neurology, Ludwig Maximilian University, Munich, Germany

**Keywords:** benign paroxysmal positional vertigo, nystagmus, posterior canal BPPV, horizontal canal BPPV, diagnostic positional tests

## Abstract

**Objective:**

Positional tests are the standard for diagnosing benign paroxysmal positional vertigo (BPPV). We evaluated the relationship between the timing of the testing during the day and the rate of pathological findings.

**Methods:**

A retrospective analysis of clinical records from 929 patients with a medical history consistent with the diagnosis of BPPV from Jan 1st, 2023 until Dec 31st, 2024 was performed. The aim was to assess timing of examinations and diagnostic findings. Morning examinations were categorized as those initiated from 8 a.m. to 12 p.m., while afternoon examinations were defined from 2 p.m. to 5 p.m. The rate of pathological positional tests was evaluated based on the observation of positional nystagmus induced by the diagnostic maneuvers.

**Results:**

A total of 929 individuals with the presumed diagnosis of BPPV were examined by diagnostic positional maneuvers. Five hundred and ninety individuals (63.51%) were examined in the morning and 339 (36.49%) in the afternoon. The positive rate of BPPV testing was 72.54% (428/590) in the morning group and 64.01% (217/339) in the afternoon group (*P* < 0.01). Subgroup-analysis by semicircular canal type showed the number of posterior semicircular canal BPPV (pcBPPV) was 308 of 590 (52.20%) in the morning and 154 of 339 (45.43%) in the afternoon, with a significant difference (*p* = 0.010). For horizontal semicircular canal BPPV (hcBPPV), the number was 115 of 590 (19.49%) in the morning and 60 of 339 (17.70%) in the afternoon (*p* = 0.080). Hourly analysis for both canal types revealed the highest positive rate (74.6%) between 8:00 a.m. and 8:59 a.m. and lowest (51.9%) between 2:00 p.m. and 2:59 p.m., with higher positive rates in the morning (*p* = 0.005). Multi-variate analysis showed a strong association with examination timing (*p* = 0.005) with no correlation between age/gender and positive testing.

**Conclusion:**

The overall positive rate for positional tests for BPPV was significantly higher in the morning than in the afternoon. Subgroup analysis showed a statistically difference for pcBPPV but not for hcBPPV. These findings hold clinical implications for optimizing examination scheduling and improving diagnostic and treatment strategies for patients with vertigo. Prospective studies are warranted to validate the reliability and validity of these observations.

## Introduction

Benign paroxysmal positional vertigo (BPPV) represents the most prevalent peripheral vestibular disease. The reported prevalence is between 10 and 140/100,000 and the lifetime prevalence is at least 2.4% (1–3). The underlying mechanisms are most often canalolithiasis and rarely cupulolithiasis (4). BPPV can be categorized into different types based on the affected semicircular canal: posterior semicircular canal BPPV (pcBPPV), horizontal semicircular canal BPPV (hcBPPV) and rare anterior semicircular canal BPPV (acBPPV) and multi-canal BPPV (5–7). The diagnosis of BPPV relies on the diagnostic positional tests, which are essential for accurate identification and management of the condition.

The Dix-Hallpike and the diagnostic Semont-test are often used to detect posterior semicircular canal BPPV (6). By changing the patient’s body position, one can observe characteristic upbeat and torsional nystagmus. The Roll test focuses on diagnosing horizontal semicircular canal BPPV, inducing horizontal nystagmus through left and right side-turning movements. These two examinations can localize the affected semicircular canal, providing direct evidence for subsequent repositioning treatment. They serve as the standard for confirming BPPV, with a sensitivity of 84–92% and a specificity of 91–96% (8).

Episodes of vertigo in BPPV are linked to the time of day, occurring more frequently after waking up in the morning and less frequently in the afternoon (9, 10). Therefore, we hypothesized that the positive rate of the diagnostic positional tests in patients with BPPV is also influenced by the time of day. In this retrospective study, we collected clinical data of patients with a medical history consistent with the diagnosis of BPPV from January 1st, 2023 to December 31st, 2024. We investigated the variation in positive rates of positional tests conducted at different times of the day. Our findings may have significant implications for improving the diagnostic rate of BPPV and deepening our understanding of its underlying mechanisms.

## Methods

### Patient selection and data collection

This was a retrospective study of outpatients treated for benign paroxysmal positional vertigo (BPPV) from 2023 to 2024 at the Eighth Affiliated Hospital of Southern Medical University (The First People’s Hospital of Shunde, Foshan). The study was approved by the Institutional Review Board of the Eighth Affiliated Hospital of Southern Medical University (The First People’s Hospital of Shunde, Foshan). The diagnostic criteria for BPPV are primarily based on the 2015 guidelines established by the International Classification of Vestibular Disorders (ICVD) committee of the Bárány Society (6). All attending physicians performed preliminary diagnoses based on the history-taking component of these criteria and conducted relevant examinations, including video-oculography. The initial dataset comprised 9,008 clinical records extracted from the Picture Archiving and Communication System (PACS), including videonystagmography (VNG) examinations and diagnostic positional maneuvers.

### Inclusion and exclusion criteria

#### Inclusion criteria

Data source: Cases were extracted from the PACS database, including patients who underwent VNG, diagnostic and therapeutic maneuvers for BPPV between January 1st, 2023, and December 31st, 2024 (*n* = 9,008).

Diagnostic confirmation: Patients with a clinical diagnosis of “BPPV,” “otolith syndrome,” “benign paroxysmal xx,” “xx semicircular canal” (regardless of the affected semicircular canal) were eligible (*n* = 1,173).

#### Exclusion criteria

Duplicate records: Fifty-five repeated VNG examinations on the same day were excluded; only the initial test results were retained (*n* = 1,118).

Central pathology: Fifty-four cases with suspected central lesions (e.g., abnormal optokinetic nystagmus, spontaneous nystagmus or documented neurological comorbidities), or cases diagnosed with chronic otitis media, Menière’s disease or vestibular migraine were excluded (*n* = 1,064).

Non-relevant specialties: Seventy-one data from emergency medicine, respiratory, and gastroenterology departments were excluded. Analysis was restricted to otolaryngology-head and neck surgery, neurology, neurosurgery, and rehabilitation medicine (*n* = 993).

Physician qualification: Only cases managed by clinicians certified in BPPV guideline-based protocols were included. Sixty-four records from uncertified clinicians were excluded (*n* = 929).

#### Statistical methods

Descriptive statistics were computed by SPSS 23.0 and GraphPad Prism 8.0. A *p* value less than 0.05 was considered statistically significant. Patient age was normally distributed (assessed by the Shapiro–Wilk test) and presented as mean ± SD. Independent samples *t*-test was performed to evaluate age differences between the study cohorts. Chi-Square test was used to assess whether there was any difference between morning and afternoon procedures. In addition, a multivariable logistic regression analysis was performed to study factors associated with nystagmus detection.

**Figure 1:**
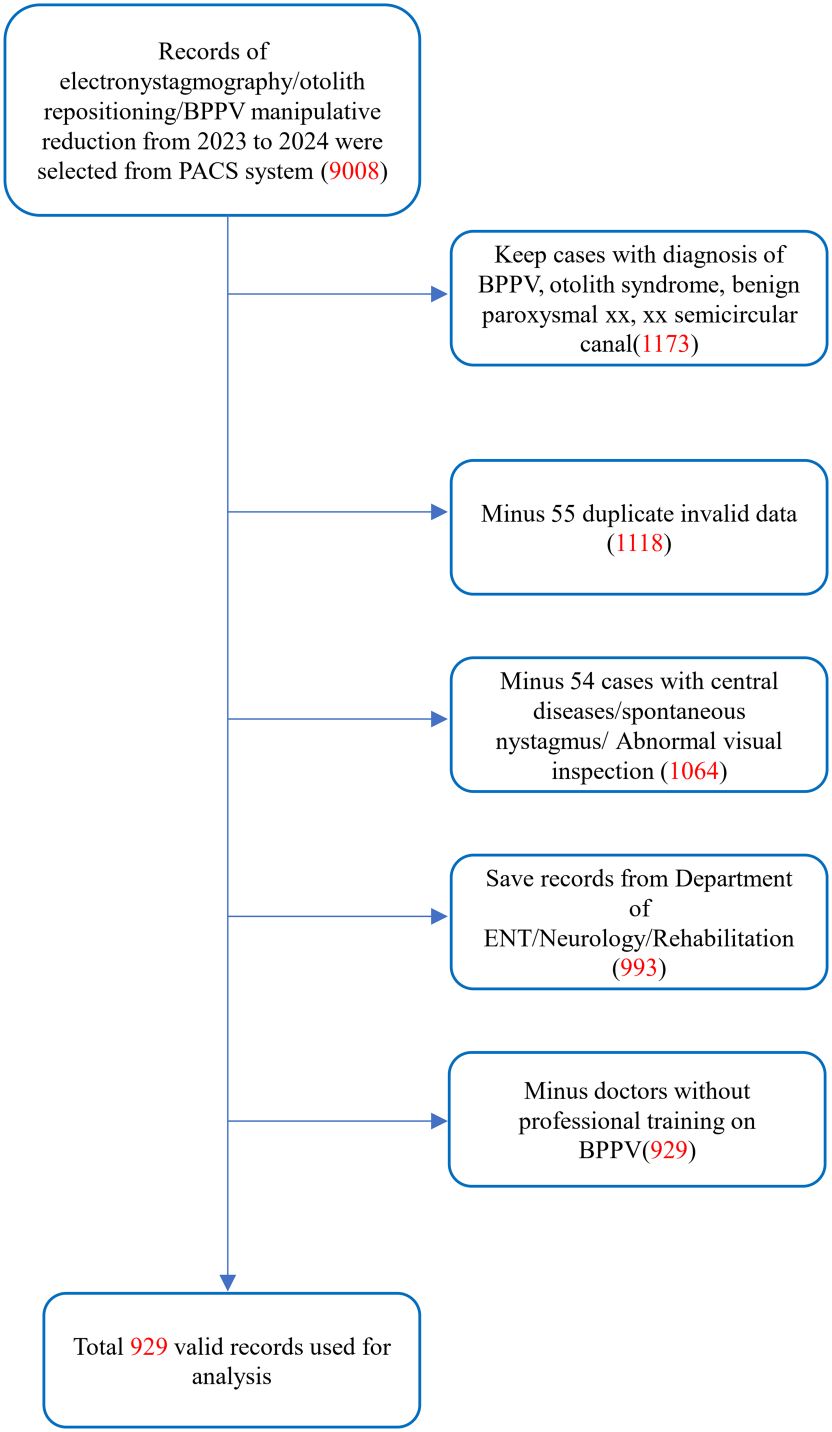
Flow chart indicating case enrollment.

The study cohort comprised over 900 cases, ensuring adequate statistical power (β > 0.8) to detect significant differences in BPPV positive rates between morning (a.m.) and afternoon (p.m.) assessments using Chi-square tests (α = 0.05, two-tailed). This sample size also satisfied the theoretical requirements for contingency table analyses, including expected cell frequency assumptions (all >5) (Figure 1).

## Results

In this study, a total of 929 patients were included in the final analysis (Table 1). Typical BPPV history (brief episodes of vertigo provoked by positional changes) was documented in all patients. More than half of patients (*n* = 590, 63.51%) received BPPV diagnostic tests in the morning and 339 (36.49%) in the afternoon. Our data showed that these cases were distributed across different age groups, with approximately 41.44% occurring in individuals under 50 years old, 45.86% in those aged 50–70 years, and 12.70% in patients over 70 years of age. The mean age of patients was similar in morning and afternoon groups. There was a significantly higher number of women (*n* = 652, 70.85%); the male-to-female ratio of patients did not differ significantly between morning and afternoon assessments (*p* = 0.602).

**Table 1:** Demographic features of positioning tests (total 929).

Factor	Morning (*n* = 590)	Afternoon (*n* = 339)	*P* value
Age (mean years, SD)	52.66, 14.82	52.44, 15.23	0.832
Age groups			
<50 years	238 (40.34%)	147 (43.36%)	0.513
50 ~ 70 years	279 (47.29%)	147 (43.36%)	
>70 years	73 (12.37%)	45 (13.27%)	
Gender			
Female (2)	418 (70.85%)	234 (69.03%)	0.602
Male (1)	172 (29.15%)	105 (30.97%)	
Detection rate			
Total positive	428 (72.54%)	217 (64.01%)	0.008
Total negative	162 (27.46%)	122 (35.99%)	
Canals			
pcBPPV	308 (71.96%)	154 (70.97%)	0.674
hcBPPV	115 (26.87%)	60 (27.65%)	
scBPPV	3 (0.70%)	3 (1.38%)	
mixBPPV	2 (0.47%)	0 (0%)	

BPPV, benign paroxysmal positional vertigo; pcBPPV, posterior semicircular canal BPPV; hcBPPV, horizontal semicircular canal BPPV; scBPPV, superior semicircular canal BPPV; mixBPPV, mixed semicircular canal BPPV.

*P* values correspond to *t*-test for age and Chi-square test otherwise.

The rate of positive positional tests for all types of BPPV-like patients was higher in the morning group compared to the afternoon group (428 out of 590, 72.54% vs. 217 out of 339, 64.01%, *P* < 0.01). Statistical analysis revealed a significant diurnal variation in the overall detection rate of BPPV-related nystagmus (*p* = 0.008), with notable differences observed between morning and afternoon examinations. There was no statistically significant difference in the positive detection rates of BPPV among the various canal types (*p* = 0.674).

In a subgroup analysis, the positive rate of BPPV positional tests between posterior semicircular canal BPPV (pcBPPV) and horizontal semicircular canal BPPV (hcBPPV) across morning and afternoon assessments was also evaluated (Table 2). It showed that the number of pcBPPV was 308 of 590 (52.20%) in the morning and 154 of 339 (45.43%) in the afternoon. For hcBPPV, the number was 115 of 590 (19.49%) in the morning and 60 of 339 (17.70%) in the afternoon. This analysis showed a significant difference in pcBPPV (*p* = 0.010), whereas no statistically significant difference was observed in hcBPPV (*p* = 0.077). Due to the limited number of cases involving anterior semicircular canal BPPV (acBPPV) and mixed-type BPPV (mixBPPV), these subgroups were not analyzed.

**Table 2:** Chi-square test of pcBPPV and hcBPPV (total number of pcBPPV: 462, hcBPPV: 175).

Type	Morning (*n* = 590)	Afternoon (*n* = 339)	*P* value
pcBPPV	308 (52.20%)	154 (45.43%)	0.010
hcBPPV	115 (19.49%)	60 (17.70%)	0.077
Total negative	162 (27.46%)	122 (35.99%)	

The outpatient clinic was open from 8:00 a.m. to 12:00 p.m. and 2:00 p.m. to 5:00 p.m. Therefore, we chose data from these hours. In the univariate, hour-by-hour analysis, the mean percentage of BPPV detected in every hour ranged from a high of 74.6% between 8:00 a.m. and 8:59 a.m. to a low of 51.9% between 2:00 p.m. and 2:59 p.m. There was a statistically significant difference between morning and afternoon (Table 3, *p* = 0.005) for the positive rate of positional tests. Additionally, the differences in the positive rates of pcBPPV across time periods are statistically significant (*p* = 0.0099), whereas hcBPPV show no significant difference (*p* = 0.161).

**Table 3:** Hourly variation in BPPV canal involvement.

Category	Hour of the day								*P* value
	8:00–8:59	9:00–9:59	10:00–10:59	11:00–12:00	14:00–14:59	15:00–15:59	16:00–17:00	Total	
Patients	59	143	172	216	77	112	150	929	
pcBPPV	33	69	91	115	26	56	72	462	0.010
hcBPPV	10	34	31	40	14	24	22	175	0.161
Positive	44	105	123	159	40	80	94	645	0.005
Negative	15	38	49	57	37	32	56	284	
Positive %	74.6	73.4	71.5	73.6	51.9	71.4	62.7	69.4	

*P* values obtained by Chi-square test statistic for categorical variables.

The data in Table 4 indicate that there was no statistically significant difference in the nystagmus positivity rate between the four examiners in the morning and afternoon (*p* > 0.05). However, the overall data show that the nystagmus positivity rate in the morning was significantly higher than in the afternoon (*p* = 0.008).

**Table 4:** Differences among examiners.

Technicians	Seniority/years of service	Morning (*n* = 590)	Afternoon (*n* = 339)	*P* value
T1	6	282 (68.44%)	150 (63.33%)	0.286
T2	12	139 (81.29%)	94 (70.21%)	0.058
T3	11	126 (71.43%)	72 (59.72%)	0.116
T4	7	43 (74.42%)	23 (56.52%)	0.170
T1 + T2 + T3 + T4		590 (72.54%)	339 (64.01%)	0.008

*P* values correspond to Chi-Square test.

T, technician.

In parentheses: The BPPV diagnostic test positivity rate was (XX%).

After adjusting for age, gender, and examiner, logistic regression analysis revealed that the time of consultation was significantly associated with the presence of nystagmus (adjusted OR = 1.628, 95% CI: 1.220 to 2.172; *P* < 0.01) (Table 5).

**Table 5:** Factors associated with vertigo: a multivariable logistic regression analysis.

Factor	OR (95% CI)	*P* value
Age	1.004 (0.994–1.013)	0.437
Female	0.923 (0.676–1.260)	0.613
Technician		
T1	Ref	
T2	1.681 (1.168–2.418)	0.005
T3	1.035 (0.721–1.486)	0.852
T4	1.047 (0.598–1.833)	0.874
Time (a.m./p.m.)	1.628 (1.220–2.172)	<0.01

T, Technician.

## Discussion

The major findings of this retrospective analysis of 929 patients with BPPV are as follows: First, the positive rate of positional tests in all patients with BPPV was significantly higher in the morning group (72.54%) compared to the afternoon group (64.01%, *p* < 0.05). Second, the subgroup analysis comparing positive diagnostic tests in the pcBPPV and hcBPPV revealed that for PC only there was a statistical difference with a trend in the patients with hcBPPV. Third, there were no significant differences in gender or age between morning and afternoon cases, allowing these variables to be excluded as confounding factors.

The difference in BPPV positional tests rates between morning and afternoon may be influenced by variations in patient activity patterns. In canalolithiasis, BPPV is associated with otolith displacement (4, 11), where changes in the position of the semicircular canals during head movements cause traction and pressure from endolymph fluid (12, 13). This leads to the displacement of hair cells in the crista ampullaris, triggering vertigo and nystagmus. Common triggers include lying down, sitting up, and turning over in bed, often resulting in vertigo episodes in the morning. A study found that 51.6% of vestibular disorder patients experienced vertigo symptoms in the morning (14). Over half of patients experienced vertigo upon waking, suggesting a link to slow otolith accumulation during sleep (10). Position during bedrest is related to the canal affected in posterior semicircular canal BPPV (15). It was also suggested that otoliths detach and fall into the semicircular canals during sleep (16). This is in agreement with the observation that reduced mobility and longer bedrest are risk factors for BPPV (17). Based on biophysical studies, it has been proposed that nighttime aggregation of otoconia amplifies their effect during morning head movements compared to individual crystals (12). Thus, it is likely that prolonged nighttime recumbency leads to otoconia aggregation, particularly in the posterior semicircular canal, which can be displaced by sudden morning movements. Posterior canal BPPV has a higher incidence than hcBPPV, primarily associated with anatomical structure, biomechanical characteristics, and physiological functions. The specific causes are analyzed as follows: In daily postures (e.g., standing upright, lowering the head, or lying on one side), the posterior semicircular canal is more prone to being in a “gravity-dependent position.” When otoliths detach from the utricle, they are more likely to fall into and remain trapped within the posterior semicircular canal due to gravitational forces. The posterior semicircular canal primarily senses vertical rotational movements (e.g., lowering or raising the head) and is more closely associated with frequent postural changes (e.g., bending over or getting up).

In contrast, the horizontal semicircular canal mainly perceives horizontal rotations (e.g., head turning), with relatively lower functional activation frequency. Even when otoliths accumulate, symptoms may be subtle and thus underestimated. Morning positional tests may also detect more BPPV cases due to inherent biological rhythms. Studies in animals and humans (18) have demonstrated anatomical and functional connections between the vestibular nuclei and the circadian rhythm system, supporting the hypothesis that vestibular function exhibits circadian rhythmicity. Research indicates that the behavior of endolymph may contribute to the higher morning positive detection rates of BPPV. It was also shown that otoconia, composed of calcium carbonate crystals formed on a protein scaffold, are primarily influenced by the calcium ion concentration in the endolymph (19). Normal endolymph, with a calcium concentration of 20 μM, dissolves otolith fragments rapidly; however, dissolution decreases when calcium levels rise above this threshold. It was also hypothesized that shallow breathing during sleep can induce mild respiratory acidosis and decrease blood pH (20). This nocturnal low-pH environment may make otoconia more susceptible to corrosion, potentially triggering BPPV onset in the morning.

On the other hand, physical activity during the day may disperse otoconia which is in line with the observation of fatigability in BPPV (4, 21): aggregates of otoconia may gradually disintegrate during physical activity and head position changes. This is also supported by biophysical experiments (12) showing that small particles induce minimal displacement of endolymph and the otolithic membrane, with a reduction of even failing to evoke positional nystagmus. Our study found that the positive rate of BPPV positional tests reached its lowest point between 2:00 p.m. and 2:59 p.m., followed by a slight rebound (still lower than morning rates) between 3:00 p.m. and 4:00 p.m. This pattern may be linked to the napping habits of the Chinese population. The nadir at 2:00 p.m.–2:59 p.m. could be due to patients consulting in the late morning and then undergoing examinations in the afternoon after waiting for an extended period, potentially resulting in otoconia fatigue from repeated postural changes. Those examined after 3:00 p.m., who rested in a reclined position during their noon break, may have experienced gravitational re-accumulation of otoliths in the semicircular canals during their naps. This positional shift likely heightened their sensitivity to positional stimulation, contributing to the observed rebound in positive test rates.

Further, previous studies have demonstrated a risk of false-negative diagnoses in the Dix-Hallpike maneuver (22–26). Clinical observations revealed that 10–20% of patients with initially negative results were later confirmed positive upon repeated testing (24). This phenomenon may be related to the formation of otolith debris clusters, which enhances stimulation of the crista ampullaris during postural changes. This supports the recommendation of repetitive diagnostic maneuvers to improve the diagnostic rates of BPPV (8).

Moreover, our data analysis revealed that all technicians did not exhibit any significant time effect. All four technicians underwent standardized training and adhered to quality standards for nystagmus examination, ensuring consistency in testing techniques. Technician 2’s longer work experience may have influenced the positive rate, while the smaller sample sizes for technicians 3 and 4 could have introduced bias. Therefore, this study does not provide evidence that examiner factors are the primary cause of reduced afternoon positive rates for BPPV. Further validation is needed through stratified analysis, balanced sample sizes, and repeated measures designs.

## Limitations

First, this was a single-center, retrospective analysis. Second, confounding factors were not systematically evaluated. Third, the sample sizes for the subgroup of patients with HC BPPV was quite small. Future research should employ a prospective design.

## Conclusion

In conclusion, the positive rate for positioning tests for BPPV was significantly higher in the morning than in the afternoon in our study. We hypothesize that this phenomenon can be explained by biophysical studies which show that the impact of larger aggregates of otoconia on endolymphatic flow is greater than that of single crystals. From a clinical point of view, these findings highlight the importance of optimizing examination schedules to improve diagnostic and treatment strategies for BPPV patients. Prioritizing morning examinations, especially during early hours, could enhance diagnostic efficiency, reduce medical waste and optimize medical resource allocation. Prospective studies are needed to validate the reliability and validity of these observations.

## Data Availability

The datasets analyzed during the current study are not publicly available due to privacy concerns of patients but are available from the corresponding author on reasonable request.

## References

[1] von Brevern M Radtke A Lezius F Feldmann M Ziese T Lempert T. Epidemiology of benign paroxysmal positional vertigo: a population based study. J Neurol Neurosurg Psychiatry. (2007) 78:710–5. doi: 10.1136/jnnp.2006.100420, PMID: 17135456 PMC2117684

[2] van der Zaag-Loonen HJ van Leeuwen RB Bruintjes TD van Munster BC. Prevalence of unrecognized benign paroxysmal positional vertigo in older patients. Eur Arch Otorrinolaringol. (2015) 272:1521–4. doi: 10.1007/s00405-014-3409-4, PMID: 25488279

[3] Neuhauser HK. The epidemiology of dizziness and vertigo. Handb Clin Neurol. (2016) 137:67–82. doi: 10.1016/B978-0-444-63437-5.00005-4, PMID: 27638063

[4] Brandt T Steddin S. Current view of the mechanism of benign paroxysmal positioning vertigo: cupulolithiasis or canalolithiasis? J Vestib Res. (1993) 3:373–82. 8275271

[5] Bhattacharyya N Gubbels SP Schwartz SR Edlow JA El‐Kashlan H Fife T. Clinical practice guideline: benign paroxysmal positional vertigo (update). Otolaryngol Head Neck Surg. (2017) 156:S1–S47. doi: 10.1177/0194599816689667, PMID: 28248609

[6] von Brevern M Bertholon P Brandt T Fife T Imai T Nuti D. Benign paroxysmal positional vertigo: diagnostic criteria. J Vestib Res. (2015) 25:105–17. doi: 10.3233/VES-150553, PMID: 26756126

[7] Power L Murray K Szmulewicz DJ. Characteristics of assessment and treatment in benign paroxysmal positional Vertigo (BPPV). J Vestib Res. (2020) 30:55–62. doi: 10.3233/VES-190687, PMID: 31839619 PMC9249279

[8] Evren C Demirbilek N Elbistanlı MS Köktürk F Çelik M. Diagnostic value of repeated dix-Hallpike and roll maneuvers in benign paroxysmal positional vertigo. Braz J Otorhinolaryngol. (2017) 83:243–8. doi: 10.1016/j.bjorl.2016.03.007, PMID: 27170347 PMC9444768

[9] Valko Y Werth E Imbach LL Valko PO Weber KP. The eyes wake up: screening for benign paroxysmal positional vertigo with polysomnography. Clin Neurophysiol. (2020) 131:616–24. doi: 10.1016/j.clinph.2019.12.002, PMID: 31972505

[10] Ichijo H. Onset time of benign paroxysmal positional vertigo. Acta Otolaryngol. (2017) 137:144–8. doi: 10.1080/00016489.2016.1221130, PMID: 27577049

[11] Brandt T Steddin S Daroff RB. Therapy for benign paroxysmal positioning vertigo, revisited. Neurology. (1994) 44:796–800. doi: 10.1212/WNL.44.5.796, PMID: 8190277

[12] Obrist D Hegemann S Kronenberg D Häuselmann O Rösgen T. *In vitro* model of a semicircular canal: design and validation of the model and its use for the study of canalithiasis. J Biomech. (2010) 43:1208–14. doi: 10.1016/j.jbiomech.2009.11.027, PMID: 20035941

[13] Silva C Amorim AM Paiva A. Benign paroxysmal positional vertigo--a review of 101 cases. Acta Otorrinolaringol Esp. (2015) 66:205–9. doi: 10.1016/j.otorri.2014.09.003, PMID: 25865125

[14] Ichijo H. Analysis of 30 patients with cupulolithiasis of the posterior semicircular canal. Eur Arch Otorrinolaringol. (2023) 280:599–603. doi: 10.1007/s00405-022-07508-2, PMID: 35759045

[15] Lopez-Escamez JA Gámiz MJ Fiñana MG Perez AF Canet IS. Position in bed is associated with left or right location in benign paroxysmal positional vertigo of the posterior semicircular canal. Am J Otolaryngol. (2002) 23:263–6. doi: 10.1053/ajot.2002.124199, PMID: 12239689

[16] Shigeno K Ogita H Funabiki K. Benign paroxysmal positional vertigo and head position during sleep. J Vestib Res. (2012) 22:197–203. doi: 10.3233/VES-2012-0457, PMID: 23142834

[17] Kim H Park J Kim J. Update on benign paroxysmal positional vertigo. J Neurol. (2021) 268:1995–2000. doi: 10.1007/s00415-020-10314-7, PMID: 33231724 PMC7684151

[18] Martin T Zouabi A Pasquier F Denise P Gauthier A Quarck G. Twenty-four-hour variation of vestibular function in young and elderly adults. Chronobiol Int. (2021) 38:90–102. doi: 10.1080/07420528.2020.1835941, PMID: 33317340

[19] Zucca G Valli S Valli P Perin P Mira E. Why do benign paroxysmal positional vertigo episodes recover spontaneously? J Vestib Res. (1998) 8:325–9. doi: 10.3233/VES-1998-8404, PMID: 9652482

[20] Han D Kim D. The evolutionary hypothesis of benign paroxysmal positional vertigo. Med Hypotheses. (2020) 134:109445. doi: 10.1016/j.mehy.2019.109445, PMID: 31669757

[21] von Brevern M Bertholon P Brandt T Fife T Imai T Nuti D. Benign paroxysmal positional vertigo: diagnostic criteria consensus document of the Committee for the Classification of vestibular disorders of the Barany society. Acta Otorrinolaringol Esp (Engl Ed). (2017) 68:349–60. doi: 10.1016/j.otorri.2017.02.007 29056234

[22] Norre ME. Diagnostic problems in patients with benign paroxysmal positional vertigo. Laryngoscope. (1994) 104:1385–8. doi: 10.1288/00005537-199411000-00012, PMID: 7968169

[23] Kramer PD Kleiman DA. Dix-Hallpike maneuver results are not influenced by the time of day of the test. Acta Otolaryngol. (2005) 125:145–7. doi: 10.1080/00016480410023065, PMID: 15880944

[24] Viirre E Purcell I Baloh RW. The dix-Hallpike test and the canalith repositioning maneuver. Laryngoscope. (2005) 115:184–7. doi: 10.1097/01.mlg.0000150707.66569.d4, PMID: 15630391

[25] Whitney SL Marchetti GF Morris LO. Usefulness of the dizziness handicap inventory in the screening for benign paroxysmal positional vertigo. Otol Neurotol. (2005) 26:1027–33. doi: 10.1097/01.mao.0000185066.04834.4e, PMID: 16151354

[26] Hilton M Pinder D. The epley manoeuvre for benign paroxysmal positional vertigo--a systematic review. Clin Otolaryngol Allied Sci. (2002) 27:440–5. doi: 10.1046/j.1365-2273.2002.00613.x, PMID: 12472509

